# Morphology and Physicochemical Properties of Branched Polyurethane/Biopolymer Blends

**DOI:** 10.3390/polym12010016

**Published:** 2019-12-19

**Authors:** Joanna Brzeska, Agnieszka Tercjak, Wanda Sikorska, Marek Kowalczuk, Maria Rutkowska

**Affiliations:** 1 Department of Commodity Industrial Science and Chemistry, Gdynia Maritime University, 83 Morska Street, 81-225 Gdynia, Poland; m.rutkowska@wpit.umg.edu.pl; 2 University of the Basque Country (UPV/EHU), Department of Chemical and Environmental Engineering, Group ‘Materials+Technologies’ (GMT), Plaza Europa 1, 20018 Donostia-San Sebastián, Spain; agnieszka.tercjaks@ehu.eus; 3 Centre of Polymer and Carbon Materials, Polish Academy of Sciences, 34 M. Curie-Sklodowska Street, 41-819 Zabrze, Poland; wsikorska@cmpw-pan.edu.pl (W.S.); marek.kowalczuk@cmpw-pan.edu.pl (M.K.)

**Keywords:** branched polyurethanes, synthetic polyhydroxybutyrate, biopolymers, chemical structure, thermal and mechanical properties, surface morphology, contact angle

## Abstract

The aim of this study is the analyze the structure of branched polyurethanes based on synthetic poly([R,S]-3-hydroxybutyrate) and their blends with biopolymers and montmorillonite. The properties which would predict the potential susceptibility of these materials to degradation are also estimated. Fourier-transform infrared spectroscopy with attenuated total reflection analysis shows that poly([d,l]-lactide) is on the surfaces of polyurethanes, whereas chitosan and starch are included inside the blend network. Atomic force microscopy images have shown that the surfaces of investigated samples are heterogenous with the formation of spherulites in case of pure polyurethanes. The presence of biopolymers in the blend reduced the crystallinity of polyurethanes. Thermal stability of blends of polyurethanes with poly([d,l]-lactide) and polysaccharides decreased in comparison to pure polyurethanes. Although the tensile strength is reduced after the blending of polyurethanes with biopolymers, the elongation at break increased, especially in the case of polyurethane/poly([d,l]-lactide) blends. The presence of polysaccharides in the obtained blends caused the significant reduction of contact angle after one minute from water drop immersion. This hydrophilizing effect is the highest when montmorillonite has been incorporated into the chitosan blend. The estimated properties of the obtained materials suggest their potential sensitivity on environmental conditions.

## 1. Introduction

Taking into consideration the fact that undegradable polyurethane (PUR) waste influences the natural environment, it is advisable to find a solution to this problem. Among all propositions, polyurethane modification using natural components seems to be the most reasonable one. It is difficult to replace synthetic polymers with natural equivalents due to typically worse properties of such materials, especially in terms of mechanical properties. However, introducing the natural compounds into a polymer structure or polymer network means the properties of pure polymers could be preserved while the material will gradually degrade at the end of the life cycle. Natural substrates usually used for polyurethanes modification are vegetable oils, polysaccharides, proteins, plants fibres, poly(hydroxy acids) and poly(lactide acid) [1,2,3]. The presence of chitosan (Ch) and starch (St) in polyurethane network causes increasing the sorption properties and hydrophilicity of pure polymer [4,5]. The degradability of polyurethanes can be also affected through modification with the synthetic polymers that are equivalents of natural polymers, such as poly([d,l]-lactide) (PLA) and poly([R,S]-3-hydroxybutyrate) (R,S-PHB) [6].

Immersing the natural substrates or their synthetic analogues inside the synthetic polymer network enables the migration of water molecules between macrochains and facilitates the degradation of polymer chains. It is giving a chance that also synthetic polymer chains will be biodegraded, without producing the very unwanted microplastics. For polyurethane synthesis, it is important that the substrates are susceptible to degradation under the influence of environmental factors without producing toxic products, such as linear diisocyanate (like 4,4′-methylene dicyclohexyl diisocyanate (H_12_MDI)) and oligoesters (like polycaprolactone). Moreover, the penetration of water with other degradation factors into the structure of the polymer is easier in the absence of cross-linking, both chemical and physical.

Branched polyurethanes are polymers which have low degree of chemical crosslinking, but on the other hand, the presence of branches does not allow the formation of too many hydrogen bonds. This facilitates the migration of water molecules into the polymer structure. The sorptive properties of aliphatic branched polyurethanes can be controlled by operating the number of branch nodes and the appropriate structure of soft segments [7].

Sometimes, a significant improvement of polyurethane composite or blend properties can be obtained after introducing nanoclay into their structure [8]. Nanoclays can be organized into illite, halloysite, bentonite, kaolinite, montmorillonite, hectorite and chlorite [9]. Montmorillonite (Mt) is the most commonly used clay to modify polymers, also for obtaining hybrid polymer composites. However, because of its hydrophilic character it has to be modified via surface compatibilization or intercalation into material more organophilic and compatible with synthetic polymers [9]. 

To our knowledge, not many papers have considered the synthesis of branched polyurethanes with synthetic, amorphous poly([R,S]-3-hydroxybutyrate) in the structure and blends of these polyurethanes with biopolymers. Often, the so-called natural polyhydroxybutyrate or its hydroxyvalerate copolymer obtained by biochemical route are used [3,10]. In the biosynthesis process, during extraction through bacterial cell walls, the natural polyhydroxybutyrate is transformed from amorphous material into crystalline one [11]. It changes its properties. In contrast, the synthetic polyhydroxybutyrate obtained from β-butyrolactone is an amorphous material, such as in living cells. This makes it susceptible to degradation under natural conditions. Our previous research indicates that linear and cross-linked polyurethanes with poly([R,S]-3-hydroxybutyrate) are slowly degraded [12,13]. 

Our far-reaching goal is to check whether the introduction of a small number of branches into the polyurethanes structure, and the blending of these polyurethanes with biopolymers will change their sensitivity to external factors. However, in our opinion, the first step is to investigate the properties that could affect the potential degradability of these materials.

In this paper the results of analysis the structure, surface topography, hydrophilicity, thermal and mechanical properties of branched polyurethanes based on synthetic poly([R,S]-3-hydroxybutyrate) and blended with chitosan, poly([d,l]-lactide) and starch are presented. The influence of organophilized montmorillonite on the properties of a PUR/Ch blend is also estimated.

## 2. Experimental

### 2.1. Materials

β-butyrolactone (Aldrich, St. Luis, MO, USA) was distilled over CaH_2_ according to [14]; 18-crown-6 complex (Fluka, Germany), 3-hydroxybutyric acid sodium salt and 2-iodoethanol (Aldrich, St. Luis, MO, USA) were used as received. Oligomerols of R,S-PHB (M_n_ 2100), polycaprolactonetriol (PCL_triol_) (M_n_ 900, Aldrich, St. Luis, MO, USA) and polycaprolactonediol (PCL_diol_) (Mn 1900, Aldrich, St. Luis, MO, USA) were dried by heating at 70 °C under reduced pressure (1.4 hPa). 4,4′-methylene dicyclohexyl diisocyanate (H_12_MDI) (Aldrich, St. Luis, MO, USA) was vacuum distilled; 1,4-butanediol (1,4-BD) (Aldrich, Steinheim, Germany) was distilled azeotropically with benzene; *N,N*-dimethylformamide (DMF) (Chempur, Gliwice, Poland) was dehydrated over diphosphorous pentoxide (P_2_O_5_) and distilled under low pressure. Catalyst tin(II) octanoate (OSn) (Alfa Aestar, Karlsruhe, Germany) was used as received. Poly([d,l]-lactide) (PLA) (M_n_ 18,000-28,000, Aldrich, Steinheim, Germany), starch (St) (pea starch, HengshuiFuqiao Starch Co., Hengshui, China) and commercial montmorillonite (Mt) (Zębiec Mining and Metal Works, Zębiec, Poland) were also used as received. Montmorillonite was organophilized with an alkylarylammonium salt containing aliphatic and aromatic substituents with several hydroxyl groups, inter-platelet distance d001 = 3.7 nm [15]. Chitosan (Ch) (M_n_ 171000, degree of deacetylation 97%, MRI Gdynia, Gdynia, Poland) was ground before adding. 

### 2.2. Methods

#### 2.2.1. ATR-FTIR Spectroscopy

Fourier-transform infrared spectroscopy (FTIR) investigations were performed using attenuated total reflection (ATR, Smart Orbit Accessory, Thermo Scientific, Madison, WI, USA) in a Nicolet 3800 FTIR spectrometer (Thermo Scientific, Madison, WI, USA). To obtain a spectrum, 32 scans were taken at an optical resolution of 4 cm^−1^. For the ATR-FTIR investigations, the materials were pressed on the diamond cell to achieve surface sensitive test results.

#### 2.2.2. Surface Topography

Atomic force microscopy (AFM) images were obtained with a Nanoscope IIIa scanning probe microscope (Multimode, Bruker, Billerica, MA, USA) under ambient conditions. Tapping mode (TM) was employed in air using an integrated tip/ cantilever (125 mm in length with ca. 300 kHz resonant frequency). Typical scan rates during recording were 0.7–1 line/s using a scan head with a maximum range of 25 × 25 μm. Some samples were cut using an ultramicrotome Leica Ultracut R with a diamond blade for obtaining the sample cross section. 

Optical microscopy (OM) micrographs were taken using a Nikon Eclipse E600W microscope (Mettler FP 82 HT, Melvile, NY, USA). The micrographs were collected with the software analySISdocu FIVE.

#### 2.2.3. Thermal Properties

Differential scanning calorimetry (DSC) measurements were performed using a Mettler Toledo DSC3+ differential scanning calorimeter (Mettler Toledo, Columbus, OH, USA). Indium and zinc were used for DSC calibration. All the investigated samples were first heated from −80 to 190 °C at 10 °C min^−1^, then cooled from 190 °C to −80 at 10 °C min^−1^, and finally, the melting was performed by heating the samples up to 190 °C at 10 °C min^−1^. All the experiments were conducted under a nitrogen flow of 20 mL/min using 5–10 mg samples in aluminum pans. The melting temperature (T_m_) corresponds to the maximum of the endothermic peak. 

The thermogravimetric analysis (TGA) was performed with a TGA/SDTA-851e equipment (Mettler Toledo, Columbus, OH, USA) under air atmosphere at a heating rate of 10 °C min^−1^ from room temperature to 800 °C.

#### 2.2.4. Contact Angle 

Contact angle measurements were carried out with sessile drop method, using Data Physics OCA 20 contact angle system (SEO Phoenix 300, Suwon, Korea) at ambient temperature. 5 mL distilled water drop was used for each measurement and the photo of the drop was taken after 0, 1 and 3 min from immersing the drop on the sample surface. Measurements for all samples were done on the side of the films exposed to air during film formation in aim to prevent the support (Teflon plates) effect. At least five measurements were made for each different system.

#### 2.2.5. Mechanical Properties

TestMachine MultiTest-1xt (Mecmesin, Slinfold, West Sussex, United Kingdom) was used to estimate of tensile strength (σ_max_) and elongation at break (ε_max_) of the obtained samples. The number of replicates for this test was 5.

## 3. Results and Discussion

### Synthesis of Polyurethanes

Oligomerole of telechelic poly([R,S]-3-hydroxybutyrate) (R,S-PHB) was obtained by anionic ring opening polymerization of ß-butyrolactone. This was initiated by 3-hydroxybutyric acid sodium salt/18-crown-6 complex at room temperature and terminated with 2-iodoethanol [16]. 

The synthesis of polyurethanes was conducted in a two-step reaction with the molar ratio of NCO:OH = 2:1 in the prepolymer step and with total NCO:OH = 1:1 [7]. The prepolymer of polyurethanes was synthesized for 3 h at 100 °C under vacuum with oligomers (PCL_diol_, PCL_triol_ and R,S-PHB) and H_12_MDI in the presence of OSn. The NCO-terminated prepolymer was dissolved in DMF, and next, its molecular weight was increased by a reaction with a chain extender (1,4-BD) for about 2 h at 60 °C. After that the polyurethane foil was formed by pouring the polymer solution on Teflon plates and heating at 120 °C in the vacuum heater for 5 h.

Chitosan, montmorillonite, poly([d,l]-lactide) and starch in DMF were added into the polyurethane solution at the end of the prepolymer extending reaction. After 15 min of stirring, the blend solution was degassed and poured on Teflon plates and heated in the same way.

Table 1 shows the samples name, the % weight of oligomerols in soft segments of PUR, and the % weight of biopolymer and montmorillonite in blend.

**Figure 1:**
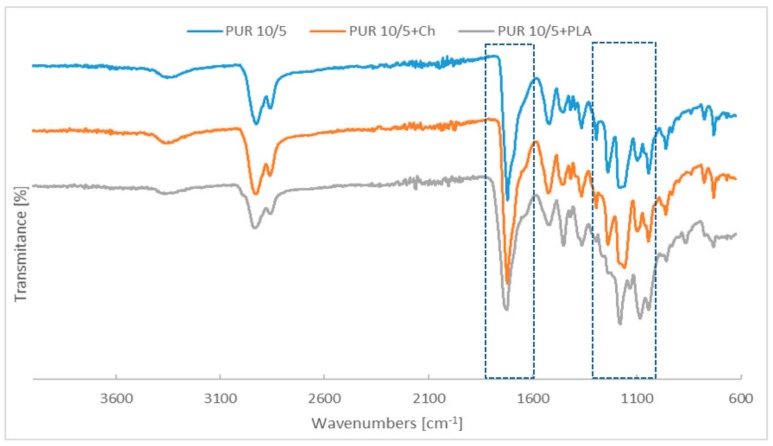
Spectra of PUR 10/5 and their blends with chitosan and poly([d,l]-lactide).

**Figure 2:**
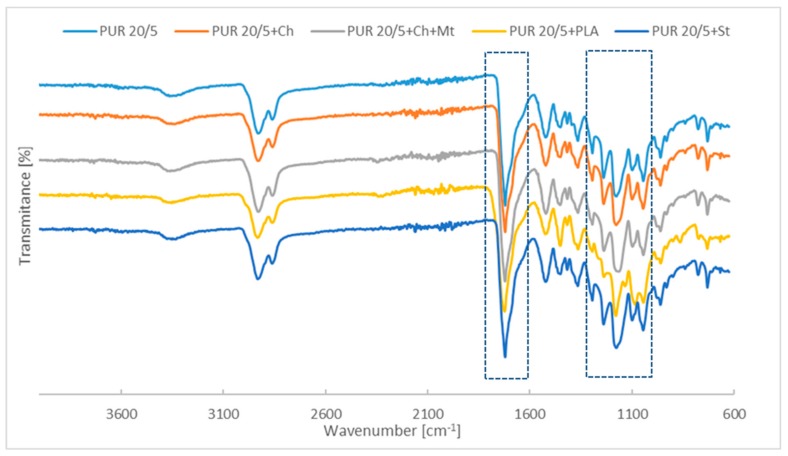
Spectra of PUR 20/5 and their blends with chitosan, chitosan with montmorillonite, poly([d,l]-lactide) and starch.

The chemical structure of the synthetized polyurethanes and their blends have been confirmed through ATR-FTIR (Figure 1 and Figure 2). 

FTIR spectra of PUR 10/5 and PUR 20/5 indicate that the synthesis reaction has been completed. The characteristic bands of urethane can be observed at Figure 1 and Figure 2: –N–H stretching vibrations at 3350–3360 cm^−1^, –C=O stretching vibrations at 1721 cm^−1^, about 1520 cm^−1^ (amide II, a combination of –N–H out-of-plane bending and –C–N stretching), and 1239 cm^−1^ (amide III, combination of –N–H bending and –C–N stretching) [17].

**Figure 3:**
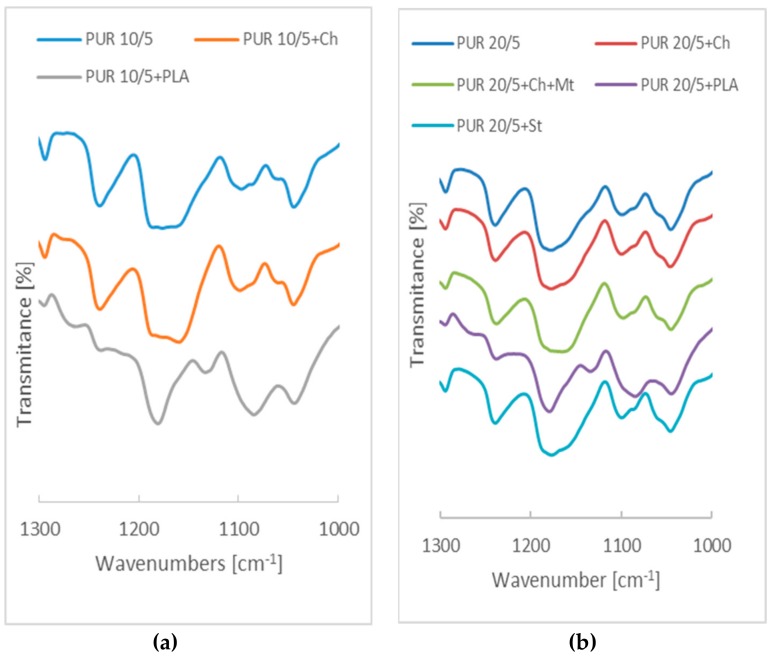
Spectra of PUR 10/5 (**a**) and PUR 20/5 (**b**) and their blends in the wavenumbers range assigned to stretching C–O bonds in soft segments.

Compared with the PUR 10/5 and PUR 20/5, the ATR-FTIR spectra of blends with chitosan, montmorillonite and starch have not been changed. However, significant changes in the polyurethane structure are visible after blending the PURs with poly([d,l]-lactide) (Figure 3). The intensity of amide II band and amide III band in PUR 10/5+PLA and PUR 20/5+PLA is lower than in pure polyurethanes.

Band of N-H stretching vibration of PUR 20/5 with higher amount of poly([R,S]-3-hydroxybutyrate) is discreetly shifted to the higher wavenumber in comparison to PUR 10/5 what suggests that the formation of hydrogen bonds between urethane groups is limited (Table S1). The probably reason is presence of later methyl group in poly([R,S]-3-hydroxybutyrate) chain what moves the chains away and hinders forming of hydrogen bonds.

Introducing of chitosan, starch and poly([d,l]-lactide) into PUR 10/5 network causes that oxygen from ester groups of soft segments is engaged in formation of hydrogen bonds with OH groups from chitosan and starch and ester groups of poly([d,l]-lactide). The result is that number of free N-H has increased (Table S1). In case of PUR 20/5 blending with biopolymers increased has caused the intensive physical interaction via hydrogen bonds between N–H of urethane and OH of polysaccharides and C=O from poly([d,l]-lactide), and in consequence, the amount of associated urethan bonds increased (wavenumber of N–H stretching vibration has decreased).

**Figure 4:**
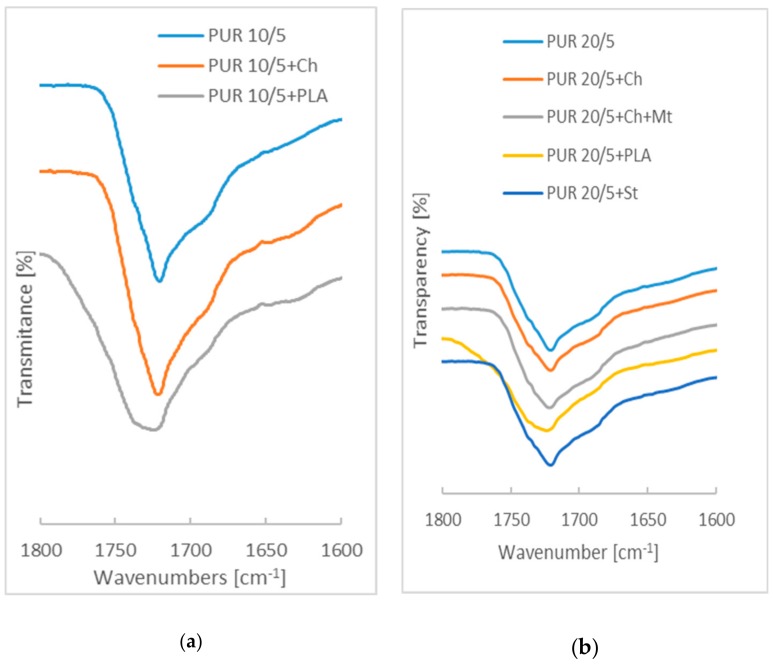
Spectra of PUR 10/5 (**a**) and PUR 20/5 (**b**) and their blends in the wavenumbers range assigned to C=O stretching vibration.

Figure 4 shows the overlapping of bands corresponding to the stretching vibrations of –C=O ester groups from polyurethane and poly([d,l]-lactide). On the spectra of PUR 10/5+PLA and PUR 20/5+PLA, the bands at about 1721 cm^−1^ overlap, with bands at 1734 cm^−1^ and 1738 cm^−1^, respectively, corresponding to ester groups of poly([d,l]-lactide) and, consequently, bands corresponding to the stretching vibrations of –C=O ester groups are moved to 1724.3 cm^−1^ and 1723.8 cm^−1^. Stretching vibration of -C=O from pure poly([d,l]-lactide) is at 1740 cm^−1^ (Supplementary Figure S1).

The main differences are observed in region of infrared absorption at 1000 cm^−1^–1300 cm^−1^. There are wide peaks at 1160 cm^−1^–1178 cm^−1^ attributed to the symmetric C–O–C stretching modes of ester groups in PURs derived from PCL and PHB chains. In the case of PURs with PLA, these bands are overleapt through sharp peak of C–O–C from ester groups of PLA.

As the FTIR investigations were performed using ATR, the changes in spectra suggest that poly([d,l]-lactide) is in the entire volume of the polyurethane network and also on its surface. Chitosan, montmorillonite, and starch are found inside the sample and there are polyurethane chains on the surface.

Taking into account the fact that glass transition temperature (T_g_) of polymers after second heating is considered as real T_g_, the data are presented in Table S2.

The glass transition temperature of the soft segments decreased after second heating of sample films in comparison to first heating. Moreover, as the samples were synthesized about two months before these analyses, their structures have changed and crystalline forms formed. However, during the cooling cycle in DSC, they could not form, so on thermograms of second heating only T_g_ is observed. The presence of a higher amount of poly([R,S-3-hydroxybutyrate) (with T_g_ about −5.0°C) in soft segments structure caused T_g_ of PUR 20/5 and its blends to be higher than for PUR 10/5 samples. It can also be seen that T_g_ of the soft segments is discreetly changed after blending polyurethanes with biopolymers (Supplementary Table S2). The increased T_g_ of PUR 20/5+Ch, PUR 20/5+Ch+Mt and PUR 20/5+St in comparison to pure PUR 20/5 may result from the extra physical crosslinking via hydrogen bonding. Meanwhile, the lowering of the glass transition temperature for the remaining samples results from the plasticizing effect of the added biopolymers.

However, the first heating scan for DSC analysis has also been interpreted since the ATR-FTIR and the morphological studies of the samples surface have been conducted on non-heated samples.

DSC thermograms after the first heating scan indicate that the obtained polyurethanes PUR 10/5 and PUR 20/5 are elastomers with the glass transition temperature of the soft segments at −24.5 °C and −18.0 °C, respectively. The small enthalpies of endothermic melting peaks at 47.5 °C and 50.4 °C indicate the low crystallinity of these polymers (Figure 2). Knowing that melting temperature of crystalline phase of PCL_diol_ is 60.1 °C and 40.7 °C for PCL_triol_ [7], these endothermic peaks on DSC thermograms correspond to the melting of soft segments of obtained polyurethanes.

Blending the synthesized polyurethanes with biopolymers caused a decrease in the glass transition temperature (Table 2). Macrochains of poly([d,l]-lactide) and starch acted as plasticizers that caused the glass transition of soft segment chains at lower temperatures. Blending PURs with particles of chitosan also reduced the T_g_ temperatures. As it was observed by Javaid’s group [18], the increase or unevenness in the distance between urethane groups through the introduction of the chitosan has decreased the H-bonding. This meant less energy for soft segments to move, so T_g_ of polyurethanes obtained with chitosan has decreased as compared to polyurethanes without chitosan. Despite the fact that Javaid’s group immersed chitosan in PURs backbone, we believe that the same relation is possible also in case of physical blending. As mentioned before, the thermograms from the second heating cycle of PUR 20/5 blends, after cooling in a short time, indicate that changes have occurred in the structure of the blends. These differences in the conditions of cooling, in comparison to the situation after sample synthesis, have caused other interactions to occur between the components of the blends. Hence the differences in the impact of biopolymers on T_g_ of polyurethanes in comparison to results of DSC second heating.

Blending polyurethanes PUR 10/5 and PUR 20/5 with biopolymers have reduced their melting enthalpy (Table 2). Although the mass of added biopolymers is small, there is a lot of them by volume. Therefore, they have a significant impact on the separation of polyurethane chains and prevent their arrangement, which could lead to the formation of crystallites. However, it should be also considered that decreasing crystallinity is only apparent. As is said above, the volume of added biopolymers is high, so the absolute number of polycaprolactone chains that can only crystallize in the soft segments in the tested blend sample is smaller than in pure polyurethanes. This could be a reason for the decreased melting enthalpy of the soft segments. Simultaneously, well-formed crystalline forms have been created in the mixtures, as is indicated by a slight shift in melting points towards higher values.

**Figure 5:**
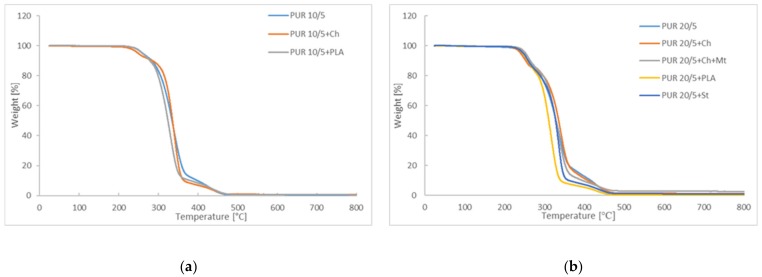
Thermogravimetric analysis thermograms of (**a**) PUR 10/5 and (**b**) PUR 20/5 and their blends.

The results of thermal degradation of polyurethanes indicate that increasing of poly([R,S]-3-hudroxybutyrate) in structure has reduced their thermal stability (Figure 5). The first mass reduction (for 1 wt.%) is observed at 244 °C and 231 °C for PUR 10/5 and PUR 20/5, respectively (Table 2). Further, blending polyurethanes with poly([d,l]-lactide)and polysaccharides caused lowering of the degradation temperature. However, adding of montmorillonite into structure of blend PUR 20/5/Ch+Mt has improved the thermal stability, what has already been observed [19,20]. PURs and their blends have thermal degraded in three stages, as is shown in Supplementary Figure S2. The weight loss at first stage for PUR 20/5+Ch+Mt appeared at temperatures higher even than for PUR 20/5 (Table 2).

Blends of polyurethanes with poly([d,l]-lactide) are the less thermally stable material. They are characterized by the fastest weight loss in the second stage of thermal degradation among all tested samples. The temperatures of the final decomposition of samples (T_f_) are very similar, and are in the range of 451–461 °C (Figure 5).

**Figure 6:**
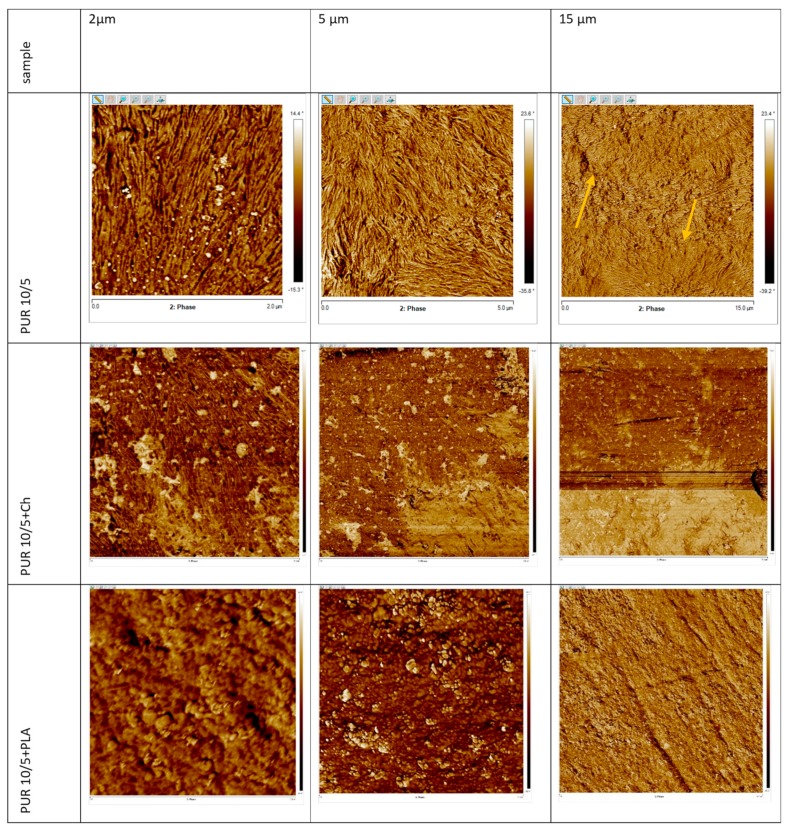
Atomic force microscopy images of surface of PUR 10/5 and its blends.

**Figure 7:**
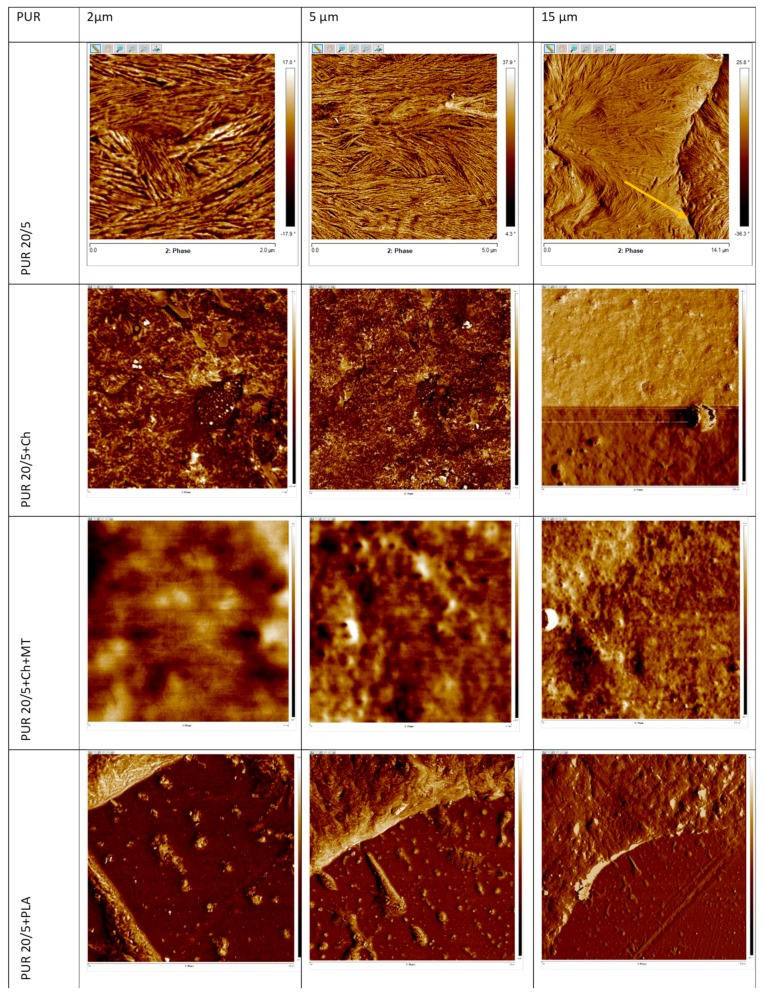
Atomic force microscopy images of surface of PUR 20/5 and its blends.

The influence of the addition of biopolymers on surface morphology of polyurethane blends was analyzed using AFM analysis (Figure 6 and Figure 7). The root mean square roughness (R_q_) and average roughness (R_a_) of AFM are given in Table 3.

The surface morphology of the PUR 10/5 and PUR 20/5 (Figure 6 and Figure 7) clearly indicates the presence of crystalline phase, which is in agreement with the results of DSC. The lamellar structures with the width 10–40 nm are probably built with the polycaprolactone chains. These lamellas arrange themselves forming spherulite forms (marked with the orange arrows in Figure 6 and Figure 7). The spherulites can be seen in the image 15 × 15µm of PUR 10/5 (Figure 6), whereas the spherulite border on surface of PUR 20/5 is visible in the image as 14 × 14µm (Figure 7). Further, cross-sectional observations of the PURs samples confirm the presence of lamellas with the width 30–50 nm and 20–30 nm for PUR 10/5 and PUR 20/5, respectively (Supplementary Figure S2).

The surfaces of PUR/biopolymer blends are different. They are heterogeneous but with low roughness (R_q_ is between 13.2 nm and 36.6 nm). Generally, except for PUR 20/5+St, the lamellar structures on blends surface are rather not observed (Figure 6). Only on surface of PUR 10/5+Ch there is a small amount of lamellae with a nanometric scale size of around 8–11 nm wide. Although the lamellar structure seen on PUR 20/5+St surface, no crystal forms are observed, which is in good agreement with low melting enthalpy (Table 2). These lamellae have not been formed into spherulites.

Interestingly, introducing the montmorillonite into PUR 20/5+Ch structure caused the surface to become smoother. This suggests an interaction between polyurethane chains, montmorillonite, and chitosan, despite that presence of hydrogen bonds between urethane and ester groups is not observed on ATR-FTIR. We concluded that presence of OH groups in montmorillonite has increased possibility of hydrogen bonding between chitosan and montmorillonite OH groups with urethan and ester groups of polyurethane. This caused chitosan particles to be precisely incorporated into the PUR network, and they do not influence surface topography.

The surface of polyurethanes observed under optical microscope is built with regular-shaped and crystalline-looking forms with a size of about 19 × 20 µm and 17 × 25 µm for PUR 10/5 and PUR 20/5, respectively (Supplementary Table S3).

Although the surface of the PURs/Ch blends is uneven, there are no inclusions of chitosan particles observed under optical microscope (Supplementary Table S3). This confirms the ATR-FTIR results, indicating that chitosan has gone deep in the polyurethane network. In comparison with PUR 10/5 and PUR 20/5, the surfaces of their chitosan blends are irregular and much less flat. The surface of PUR 10/5+Ch is built with circle forms from 12 × 12 µm to 30 × 35 µm, whereas the surface of PUR 20/5+Ch is corrugated with a lower amount of crystal forms with different sizes from 11 × 12 µm to 35 × 35 µm. The addition of the montmorilloniteinto blend network caused the surface to be a bit less waved. The surface of polyurethane blends with poly([d,l]-lactide) and starchis similar to the original polyurethane, but the visible forms have a larger size distribution.

Water contact angle measurements were performed to determine the hydrophilicity of surfaces of the films. The value of the contact angle of polyurethanes and their blends is about 80° and indicates that they are materials with low hydrophilicity. However, they are not hydrophobic (the contact angle is lower than 90° [21]) (Table 3). The high contact angle results from the presence of polycaprolactone, which is a hydrophobic material [22]. Only the contact angle of PUR 10/5+PLA is as high as 88°. This is despite the lowest surface roughness between all samples, so the reason cannot be the roughness of the surface, but the presence of poly([d,l]-lactide) on the PUR surface.The hydrophobicity of polylactide surface was observed by others [23,24]. However, the presence of a higher amount of poly([R,S]-3-hydroxybutyrate) in PUR 20/5+PLA affected the increased surface roughness. As the obtained materials are rather hydrophilic, the increase in roughness caused only slight increases in the contact angle after the PUR 20/5 blending with poly([d,l]-lactide)[21].

**Figure 8:**
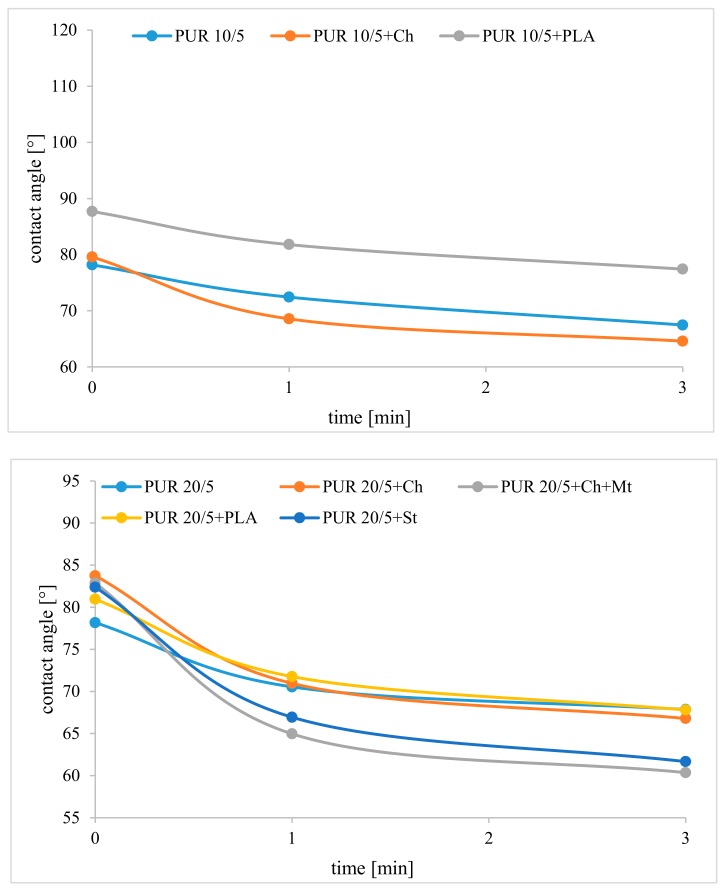
Contact angle of polyurethanes and their blends after 0, 1 and 3 min, after immersion of water drop on sample surface.

Interestingly, the contact angle rapidly decreased in the first minute after water drop immersion on the surface of blends chitosan and starch (Figure 8). Despite the fact that ATR-FTIR results indicate that both polysaccharides are immersed inside the polyurethane network, their presence caused the surface to become more hydrophilic after placing the drop. The most significant contact angle reduction is observed for PUR 20/5+Ch+Mt, which suggests that this material is likely to be most sensitive to an aqueous environment. The lowest changes in contact angle have been observed for polyurethanes with poly([d,l]-lactide). Sample photos of changing the contact angle of the surfaces are shown in Supplementary Table S2.

**Figure 9:**
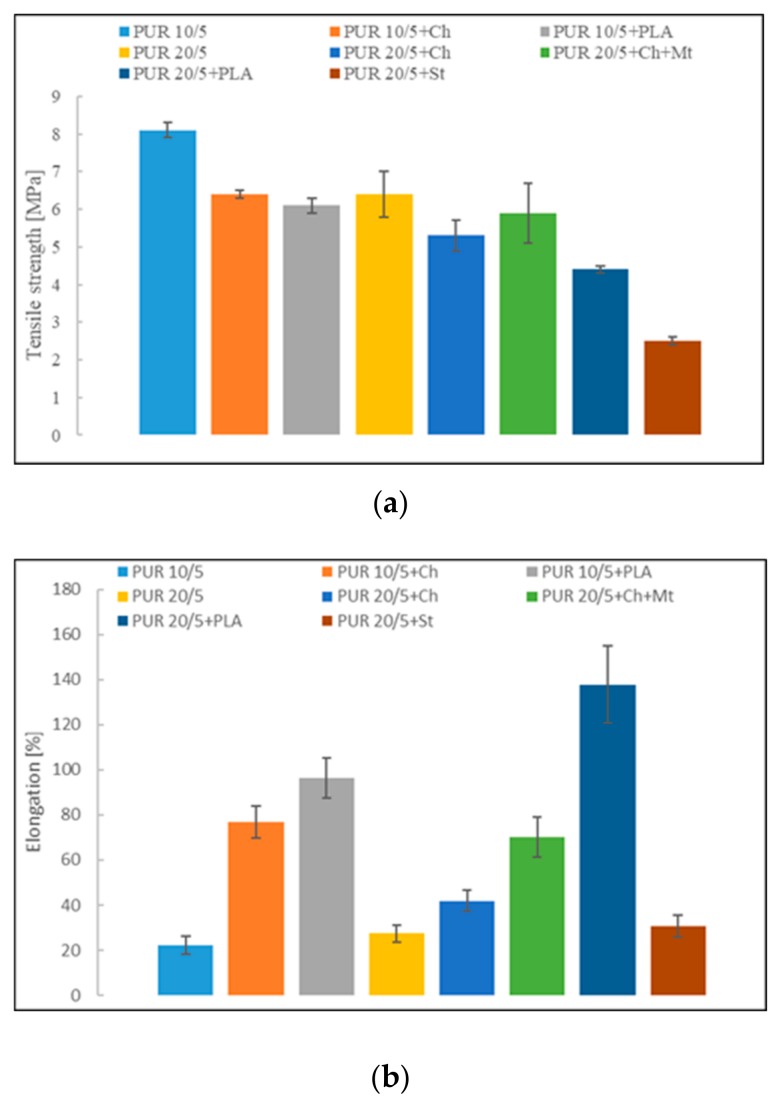
(**a**) Tensile strength and (**b**) elongation of PUR 10/5 and PUR 20/5 and their blends.

Poly([R,S]-3-hydroxybutyrate) has a secondary hydroxyl groups which hinder the polyurethane synthesis reaction. Thus, using it to obtain soft segments resulted in low mechanical properties of these polyurethanes (Figure 9a,b). Tensile strength and elongation at break are 8.1 MPa and 22.2 % and 6.4 MPa and 27.5 % for PUR 10/5 and PUR 20/5, respectively. Blending the polyurethanes with biopolymers and montmorillonite affected the decrease in tensile strength, but surprisingly, it increased elasticity. This is especially so in the case of blending with poly([d,l]-lactide). Introducing montmorillonite into the network of PUR 20/5+Ch caused a greater interaction between polyurethane chains and chitosan particles. Consequently, mechanical strength improved (Figure 9).

The interaction between chitosan and montmorillonite is described in detail in [25]. Similarly, hydrogen bonds can form between montmorillonite and urethane and ester moieties in the polyurethane chain. The dispersion of montmorillonite in the polyurethane network (AFM images did not show any agglomerates) increased the interaction surface between these groups, and thus the probability of hydrogen bonds forming.

## 4. Conclusions

Branched polyurethanes with 10 and 20 wt.% of poly([R,S]-3-hydroxybutyrate) in soft segments were blended with chitosan, montmorillonite, starch and poly([d,l]-lactide). ATR-FTIR analysis has shown that poly([d,l]-lactide) is on the surface of blends, whereas other additives of blends are inside the polyurethanes network. The surfaces of obtained polyurethanes and their blends are heterogenous, with spherules in case of pure polyurethanes, as observed under AFM and MO microscopies. The surfaces of polyurethanes blended with polysaccharides and montmorillonite became more hydrophilic one minute after placing the water drop. The highest reduction of contact angle is observed for PUR 20/5+Ch+Mt, which suggests its affinity with water. Blending the polyurethanes with biopolymers and montmorillonite causes the decrease in tensile strength, but simultaneously increases the elasticity. Presence of montmorillonite in PUR 20/5+Ch blend has increased the interaction between polyurethane and chitosan. The synthesized blends of polyurethanes based on the synthetic poly([R,S]-3-hydroxybutyrate) and a small amount of triol are low crystalline materials with an increased the affinity to water. These characteristic mean that materials will degrade in natural conditions over time dependent on the kind of added biopolymer.

Studies on the susceptibility of these polyurethane materials to degradation in the hydrolytic and oxidizing environment are currently being conducted and the results are going to be published soon. They are aimed at confirming that the obtained branched polyurethanes are going to degrade faster thanks to the introduction of biopolymers into their network.

## Figures and Tables

**Table 1:** Polyurethanes and their blends with biopolymers.

Sample	Percentage of Oligomerol in Soft Segments (wt.%)			Percentage of Biopolymer and Mt in Blend (wt.%)			
	R,S-PHB	PCL_triol_	PCL_diol_	Ch	Mt	PLA	St
PUR 10/5	10	5	85	0	0	0	0
PUR 10/5+Ch	10	5	85	2.5	0	0	0
PUR 10/5+PLA	10	5	85	0	0	5	0
PUR 20/5	20	5	75	0	0	0	0
PUR 20/5+Ch	20	5	75	2.5	0	0	0
PUR 20/5+Ch+Mt	20	5	75	2.5	1	0	0
PUR 20/5+PLA	20	5	75	0	0	5	0
PUR 20/5+St	20	5	75	0	0	0	2.5

**Table 2:** Differential scanning calorimetry and thermogravimetric analysis of polyurethanes and their blends.

Sample	T_g_ [°C]	T_g_^*^ [°C]	T_m_ [°C]	ΔH [J/g]	T_i_ [°C]	T_5%_[°C]	T_10%_ [°C]	T_f_ [°C]
PUR 10/5	−24.5	−42,2	47.5	27.4	244	266	286	454
PUR 10/5+Ch	−36.7	−45.2	52.5	13.1	231	245	279	461
PUR 10/5+PLA	−35.3	−45.0	48.9	18.5	240	257	277	454
PUR 20/5	−18.0	−36.4	50.4	25.8	231	250	264	458
PUR 20/5+Ch	−24.9	−35.1	51.8	13.1	224	238	251	455
PUR 20/5+Ch+Mt	−26.4	−34.0	50.2	11.1	242	251	263	451
PUR 20/5+PLA	−29.5	−37.8	50.1	10.1	230	244	256	453
PUR 20/5+St	−26.7	−34.8	51.1	11.9	226	245	257	456

T_g_ – glass transition temperature of soft segments from first heating scan. T_g_^*^ – glass transition temperature of soft segments from second heating scan. T_m_ – melting temperature of soft segments. ΔH – melting enthalpy of soft segments. T_i_- initial decomposition temperature of samples (representing 1% degradation of samples). T_5%_ - temperature representing 5% degradation of samples. T_10%_ - temperature representing 10% degradation of samples. T_f_ – final decomposition temperature of samples (corresponds to 1% residual dry mass of sample after degradation).

**Table 3:** Mean square roughness (R_q_) and average roughness (R_a_) of atomic force microscopy and contact angle measured immediately after drop immersion on surface of PURs and their blends.

Sample	Root Mean Square Roughness (R_q_) [nm]	Average Roughness (R_a_) [nm]	Contact Angle [°]
PUR 10/5	28	19	78.2±6.3
PUR 10/5+Ch	19	15	79.6±3.6
PUR 10/5+PLA	13	10	87.7±1.8
PUR 20/5	14	11	80.7±5.8
PUR 20/5+Ch	21	17	83.7±2.3
PUR 20/5+Ch+Mt	17	13	82.9±1.4
PUR 20/5+PLA	37	25	81.0±1.4
PUR 20/5+St	28	21	82.4±1.3

## Supplementary Materials

The following are available online at https://www.mdpi.com/2073-4360/12/1/16/s1, Figure S1: ATR-FTIR spectra of biopolymers, Figure S2: AFM pictures of cross-section PUR 10/5 (a) and PUR 20/5 (b), Table S1: ATR-FTIR bands of –N-H and –C=O stretching vibration, Table S2. Pictures of water drop on surface of selected polyurethanes and their blends, Table S3. MO images of surface of polyurethanes and their blends.
