# Supplementary material for: Morphology and Physicochemical Properties of Branched Polyurethane/Biopolymer Blends
Source: Polymers (Basel). 2019 Dec 19;12(1):16. doi: 10.3390/polym12010016 (PMC7023277; doi:10.3390/polym12010016)
Supplement: Supplementary file 1 [file polymers-12-00016-s001.pdf]

Morphology and physicochemical properties of branched polyurethane/biopolymer blends

Joanna Brzeska, Agnieszka Tercjak, Wanda Sikorska, Marek Kowalczuk, Maria Rutkowska

Supplements

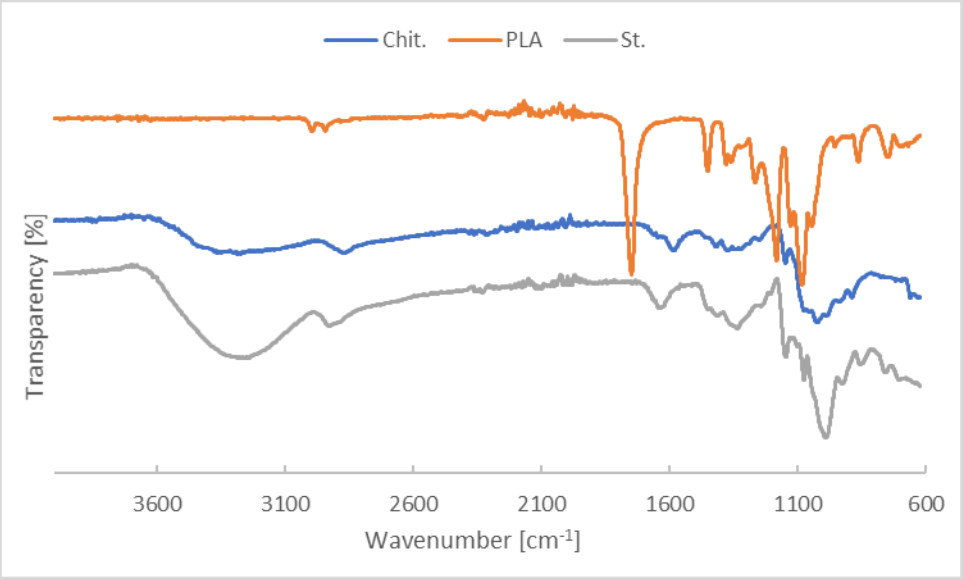

Figure S1. ATR-FTIR spectra of biopolymers.

Table S1. ATR-FTIR bands of –N-H and –C=O stretching vibration.

Sample	–N-H stretching vibration [cm^{-1}]	–C=O stretching vibration [cm^{-1}]
PUR 10/5	3351.4	1720.7
PUR 10/5+Ch	3363.3	1720.7
PUR 10/5+PLA	3357.0	1724.3
PUR 20/5	3359.4	1720.9
PUR 20/5+Ch	3356.7	1720.9
PUR 20/5+Ch+Mt	3356.5	1721.8
PUR 20/5+PLA	3352.6	1723.8
PUR 20/5+St	3351.9	1720.9

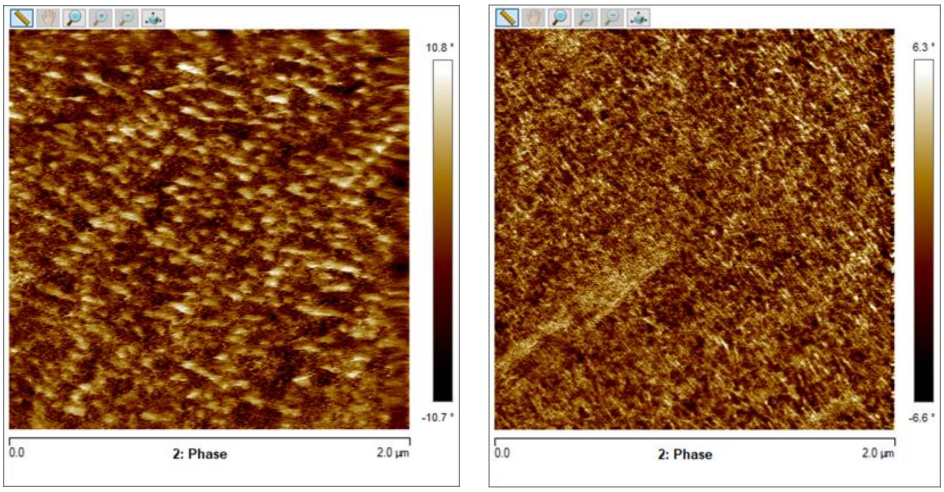

(**a**) (**b**)

Figure S2. AFM pictures of cross-section PUR 10/5 (**a**) and PUR 20/5 (**b**).

Table S2. Pictures of water drop on surface of selected polyurethanes and their blends.

Sample	Time from the immersion of water drop on polymer sample		
	0 [min]	1 [min]	3 [min]
PUR 10/5	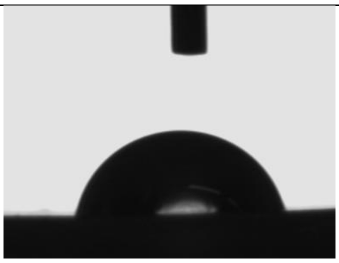	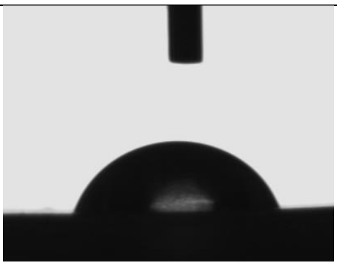	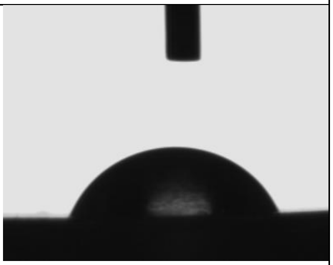
PUR 10/5 +PLA	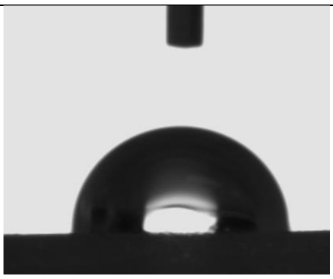	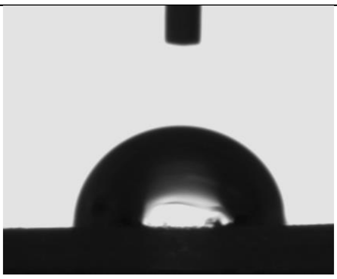	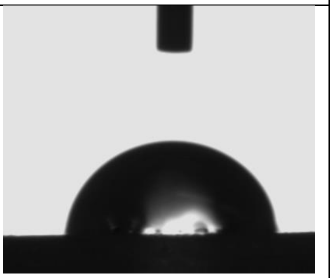
PUR 20/5 +Ch+Mt	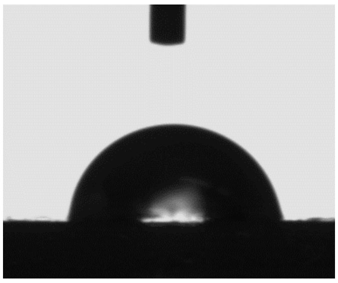	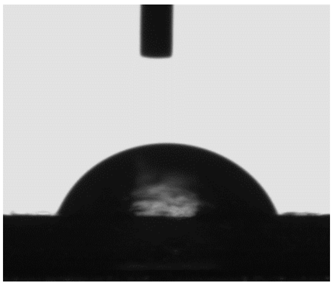	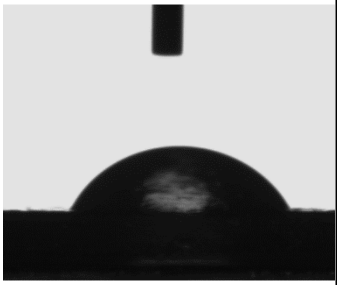

Table S3. MO images of surface of polyurethanes and their blends.

	5x	10x	20x
PUR 10/5	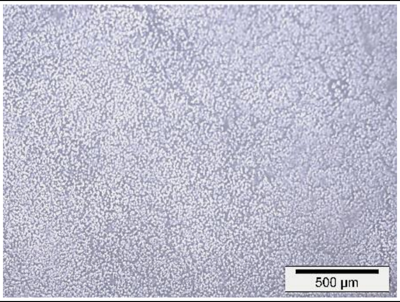	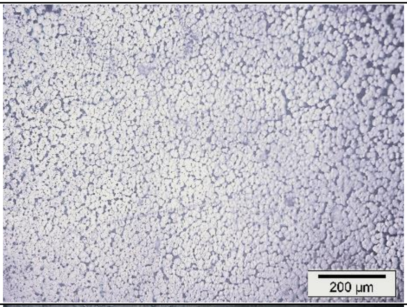	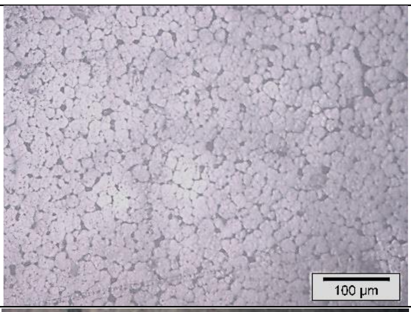
PUR 10/5+Ch	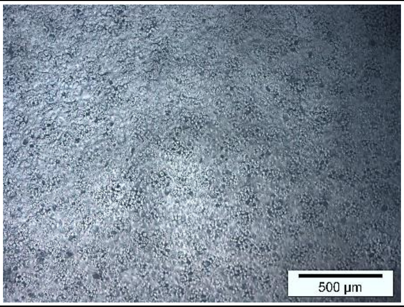	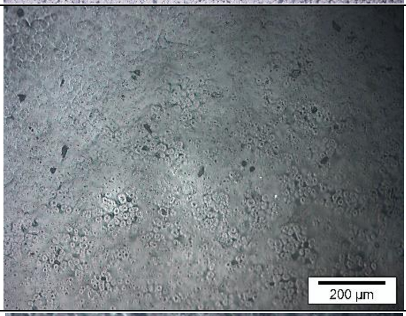	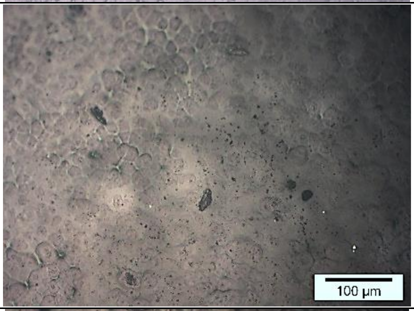
PUR 10/5+PLA	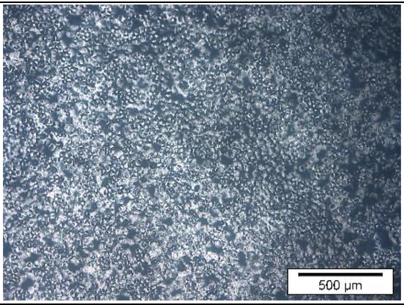	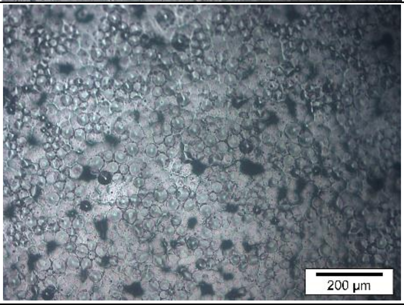	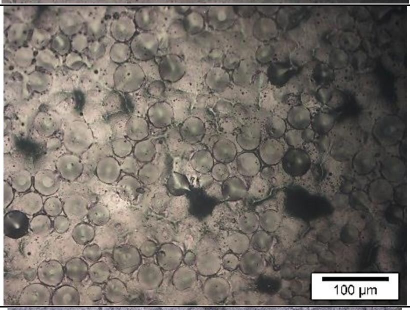
PUR 20/5	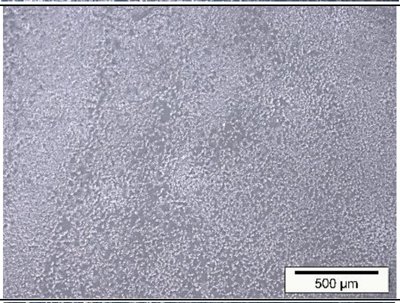	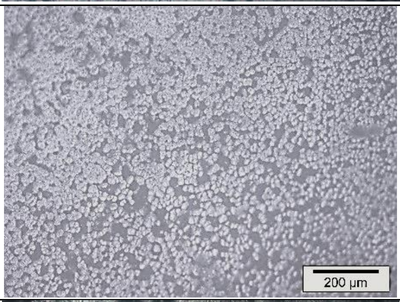	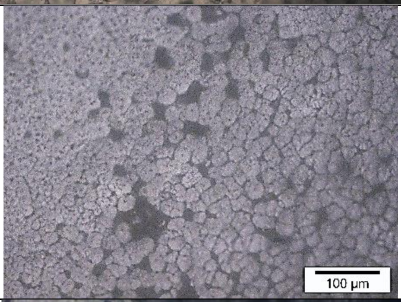
PUR 20/5+Ch	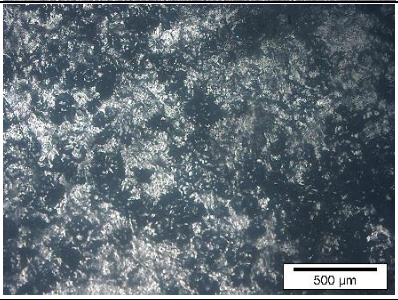	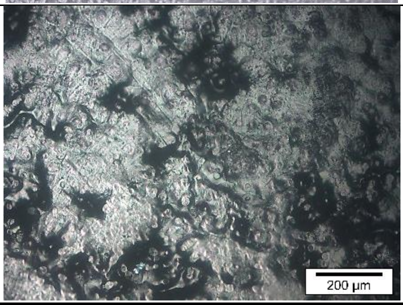	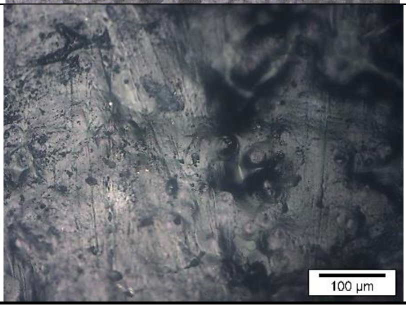

PUR 20/5+Ch+Mt	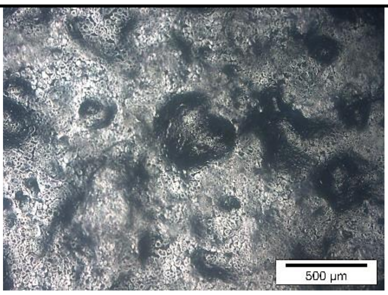	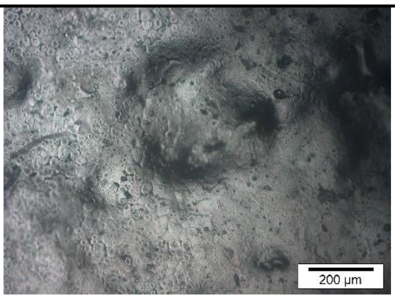	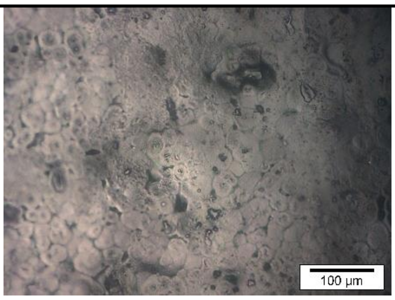
PUR 20/5+PLA	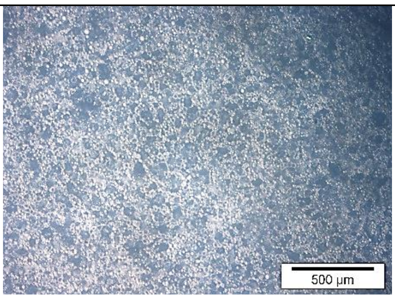	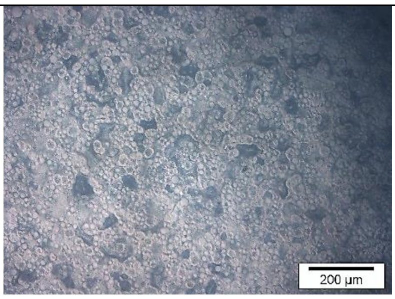	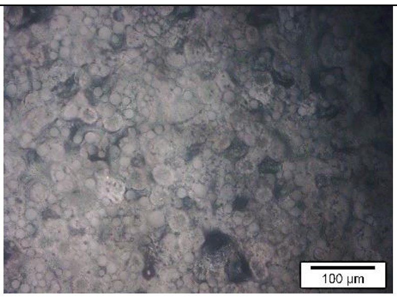
PUR 20/5+St	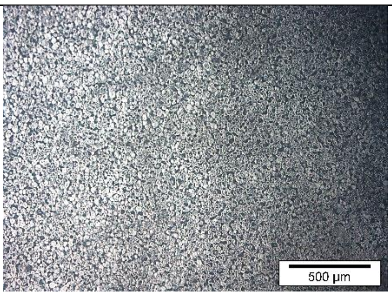	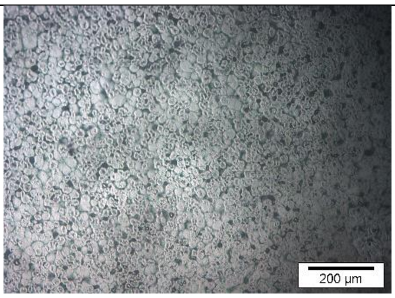	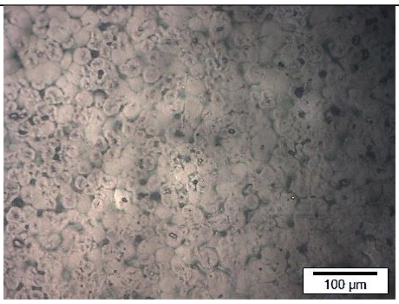
